# Fetal Sex Modulates Developmental Response to Maternal Malnutrition

**DOI:** 10.1371/journal.pone.0142158

**Published:** 2015-11-06

**Authors:** Antonio Gonzalez-Bulnes, Laura Torres-Rovira, Susana Astiz, Cristina Ovilo, Raul Sanchez-Sanchez, Ernesto Gomez-Fidalgo, Mariluz Perez-Solana, Mercedes Martin-Lluch, Consuelo Garcia-Contreras, Marta Vazquez-Gomez

**Affiliations:** 1 INIA, Madrid, Spain; 2 University of Sassari, Sassari, Italy; 3 UCM, Faculty of Veterinary, Madrid, Spain; Xavier Bichat Medical School, INSERM-CNRS - Université Paris Diderot, FRANCE

## Abstract

The incidence of obesity and metabolic diseases is dramatically high in rapidly developing countries. Causes have been related to intrinsic ethnic features with development of a *thrifty genotype* for adapting to food scarcity, prenatal programming by undernutrition, and postnatal exposure to obesogenic lifestyle. Observational studies in humans and experimental studies in animal models evidence that the adaptive responses of the offspring may be modulated by their sex. In the contemporary context of world globalization, the new question arising is the existence and extent of sex-related differences in developmental and metabolic traits in case of mixed-race. Hence, in the current study, using a swine model, we compared male and female fetuses that were crossbred from mothers with *thrifty genotype* and fathers without *thrifty genotype*. Female conceptuses evidence stronger protective strategies for their adequate growth and postnatal survival. In brief, both male and female fetuses developed a brain-sparing effect but female fetuses were still able to maintain the development of other viscerae than the brain (mainly liver, intestine and kidneys) at the expense of carcass development. Furthermore, these morphometric differences were reinforced by differences in nutrient availability (glucose and cholesterol) favoring female fetuses with severe developmental predicament. These findings set the basis for further studies aiming to increase the knowledge on the interaction between genetic and environmental factors in the determination of adult phenotype

## Introduction

The incidence of obesity and metabolic diseases like diabetes is dramatically high in rapidly developing countries such as India, Brazil, China and Middle East countries [1]. The cause, in agreement with the concept of the *Developmental Origin of Health and Disease* [2], would be related to mismatching among genetic background, nutrition of the conceptus during pregnancy, and postnatal exposure to excess of highly caloric food and scarce physical activity [3, 4]. People from developing countries are characterized by intrinsic ethnic features, with ancestors adapted to food scarcity by developing an adaptive *thrifty genotype*. A high percentage of the newborns are affected by deficiencies in prenatal growth (intrauterine growth retardation; IUGR), with this circumstance together with postnatal exposure to obesogenic environments causing childhood obesity and increased metabolic risk [4–7].

The most recent studies suggest that the adaptive response of offspring from sows exposed to nutritional challenges during pregnancy is strongly modulated by their sex, as elegantly revised by Aiken and Ozanne [8]. Females, which are more critical for the survival of the species, would have better survival and developmental traits since males are more affected by growth restriction, increased secretion of insulin and cortisol, elevated blood pressure, reduced nephron number and deficiencies in learning abilities. However, most of the data were obtained in rodents, whilst evidences in large mammals and humans are elusive.

The pig is currently considered a reliable large animal model for translational research in IUGR. In fact, swine is recognized an outstanding animal model for most of biomedical studies [9–16] since shares many anatomical (including proportional organ sizes) and physiological features (including lifestyle: diurnal rhythms, omnivorous habits, propensity to sedentary behavior and similar lipoprotein metabolism) with humans. Consequently, the pig is considered a unique model for translational research in nutrition-related pregnancy pathologies [17].

In pigs, the occurrence of IUGR after deficiencies in nutritional supply or placental development is frequent in commercial strains (e.g.: Large White or Landrace) and its developmental consequences are well-known and similar to findings in human medicine [18–21]. IUGR offspring have compromised health, reduced growth potential and high predisposition for adiposity; these effects are similar in males and females.

However, studies of our group in a different swine breed, the Iberian pig, have shown a clear sexual dimorphism in the developmental traits of the offspring exposed to nutritional deficiency [22–25]. The Iberian pig is an amenable model for studies in obesity and associated diseases [26, 27], being characterized by an adaptive *thrifty genotype* for surviving in harsh environments with food scarcity where the animals have been traditionally reared [28]. Such condition is similar to humans living in developing countries, who are characterized by intrinsic ethnic features, being descendant from ancestors adapted to food scarcity and therefore having developed *thrifty genotype* for surviving in scarce food environment [1, 3, 4, 29–33].

The Iberian male offspring affected by nutritional restriction and IUGR have reduced growth potential like the offspring from lean swine breeds [22, 25]. However, conversely, Iberian females show an early-postnatal catch-up growth, as early as during lactation, with weight and size being compensated at weaning.

Sex-related differences in developmental patterns of Iberian offspring can be found even earlier, during fetal life [24]. Iberian fetuses, having thrifty genotype, have a higher relative brain-to-body weight-ration (i.e.: prioritize brain development to the expenses of body and other organs, the so-called “brain-sparing effect”). Female Iberian fetuses are similar in size and weight to male littermates but had a significantly higher relative liver-to-body weight-ratio resembling a ‘‘liver-sparing effect” and a trend for a higher relative intestine-to-body ratio. Moreover, the availability of triglycerides, cholesterol is higher in female Iberian fetuses. These features, which were not found in fetuses from lean breeds in the same conditions, would suggest a genotype-related effect favoring female offspring in case of environmental challenges.

The scientific question arising from these results is if the sex- and breed-related differences between genotypes are driven by the maternal genotype, which may modulate the intrauterine environment, or inherent to the fetal *thrifty genotype*, which may modulate the adaptive response to such environment. Hence, in the current study, we have determined possible sex-related differences in developmental traits (absolute and relative weights of different organs and structures) and homeostasis (parameters addressing fetal stress and metabolism) in crossbred fetuses (obtained from Iberian sows, with *thrifty genotype*, inseminated with Large White semen; hence fetuses had no- *thrifty genotype*) subjected to maternal malnutrition for inducing IUGR. The results to be obtained in this study may be a first step towards the knowledge of the existence and extent of sex-related differences in developmental and metabolic traits in case of mixed-race.

## Material and Methods

### Ethics statement

The study was performed according to the Spanish Policy for Animal Protection RD1201/05, which meets the European Union Directive 86/609 about the protection of animals used in research. The experiment was specifically assessed and approved (report CEEA 2012/036) by the INIA Committee of Ethics in Animal Research, which is the named Institutional Animal Care and Use Committee (IACUC) for the INIA. The sows were housed at the animal facilities of the INIA, which meets the local, national and European requirements for Scientific Procedure Establishments.

### Animals and management

The study involved six Iberian sows, 18 months-old, that became pregnant after estrus synchronization and insemination. Estrus synchronization consisted of daily administration, for 18 consecutive days, of 20mg of the progestagen altrenogest (Regumate^®^, MSD, Boxmeer, The Netherlands), by individually top-dressing over the morning feed; the treatment was initiated irrespective of the stage of the cycle. Estrus detection was carried out twice daily, from 24 h after progestagen withdrawn, with trained sexually mature boars. Insemination with Large-White semen doses (6 × 10^9^ spermatozoa/dose) was performed 12, 24 and 36 h after estrus detection.

From starting the experimental period to Day 35 of pregnancy, the sows were fed with a standard grain-based food diet with mean values of 89.8% of dry matter, 15.1% of crude protein, 2.8% of fat and 3.00Mcal of metabolizable energy ⁄ Kg, adjusted for fulfilling individual daily maintenance requirements, based on data from the British Society of Animal Science [34].

At Day 35 of pregnancy, all the sows were weighed and their back-fat depth measured. Fat depth was determined by using a SonoSite S-Series ultrasound machine equipped with a multifrequency (5-8MHz) lineal array probe (SonoSite Inc., Bothell, WA). The probe was placed again the skin, on a point at the right side of the animal located at 4cm from the midline and transversal to the head of the last rib as determined by palpation. Concurrently, blood samples were drawn by puncture of the orbital sinus [35], into 5 ml sterile heparinized vacuum tubes (Vacutainer^™^ Systems Europe, Meylan, France). Immediately after recovery, the blood was centrifuged at 1500g for 15 min and the plasma was separated and stored into polypropylene vials at -70°C until assays.

From Days 35 to 95 of gestation, when the fetuses were sampled, sows were fed with the same standard diet but the amount of food offered to each sow was adjusted for fulfilling 50% of their daily maintenance requirements for pregnancy; such diet has been previously found to affect fetal development and to induce lower body weight in the newborns [22]. Every day, each individual food ration was weighed and given to each sow in her individual pen; hence, each female had her own diet individually adjusted to her own weight during the experimental period.

At Day 95 of pregnancy, all the sows were again weighed, measured for back-fat depth and plasma samples were obtained. Subsequently, the sows were euthanized by i.v. injection with a euthanasia solution (T-61^®^, MSD AH, Boxmeer, The Netherlands).

*Morphometric evaluation and sampling of genital tracts and fetuses*

The entire genital tracts were collected immediately after euthanasia for morphometric examination, weighing and sampling of the fetuses. The content of the uteri was exposed and the conceptuses were recorded according to their position. At once, for each conceptus, blood samples were drawn from the heart and/or umbilical cord with heparinized syringes and samples of allantoic and amniotic fluids were obtained by aspiration through the chorioallantoic and amniochorionic membranes, respectively. Blood was processed as previously described while allantoic and amniotic fluids were centrifuged at 1500g for 15 min and supernatants were stored into polypropylene vials at -20°C until assay. Finally, all the conceptuses were recovered to be sexed, measured and weighted and the empty uterus was also weighted.

### Evaluation of absolute and relative fetal measures and weights

Immediately after recovery, crown-rump length, occipito-nasal length, biparietal diameter and thoracic and abdominal circumferences were measured in all the fetuses. The total weights of each fetus and the weight of the head, trunk and main organs (brain, heart, liver, intestine, kidneys, spleen and adrenal glands) were also assessed. Afterwards, the following weight ratios were considered: weight of the fetal head relative to total weight and trunk weight; brain weight relative to head weight; weights of total viscera, brain, heart, liver, kidneys intestine, spleen and adrenal glands relative to total and trunk weight.

### Evaluation of maternal and fetal metabolic status

Parameters related to the metabolism of glucose (glucose and fructosamine) and lipids (triglycerides and cholesterol) were measured in maternal and fetal plasma and allantoic and amniotic fluids with a clinical chemistry analyzer (Saturno 300 plus, Crony Instruments s.r.l., Rome, Italy), according to the manufacturer’s instructions.

Finally, fetal stress was determined by measuring concentrations of cortisol and hydrogen peroxide (as a maker for oxidative stress) in fetal plasma by using enzymeimmunoassay kits (Demeditec Diagnostics GmbH, Kiel-Wellsee, Germany for cortisol and Abcam, Cambridge, United Kingdom for hydrogen peroxide).

### Statistical analyses

From previous studies [22], it was assumed that development of a high percentage of the fetuses was restricted by maternal undernutrition. However, there were observed some fetuses with more severe growth restriction, which we identified by the observation of an individual weight lesser than one standard deviation of the mean litter value. Effects of sex and severity of growth restriction status of the fetuses on morphological, endocrine, and metabolic features of the conceptuses were assessed by analysis of variance (one-way ANOVA) or by a Kruskall—Wallis test when a Levene’s test showed non-homogeneous variables. All results were expressed as mean ± SEM and statistical significance was accepted from P<0.05.

## Results

### Effects of nutritional restriction on maternal weight, adiposity and metabolism

The mean body-weight of the sows at Day 35, when diet-restriction started, and at Day 95 of pregnancy, when the fetuses were obtained, were 163.3±2.6 and 165.4±3.8 kg, respectively; the values for back-fat depth were also similar (42.8±2.3 and 39.0±3.6 mm for days 35 and 95, respectively). Hence, having in mind that the mean weight of the entire genital tracts with the fetuses and their annexes and fluids inside was 17.0±0.6 kg, there was a weight loss caused by the nutritional restriction of around 15kg/sow.

Food restriction also affected parameters related to glucose and lipid metabolism (Table 1). The values for glucose and total, LDL and HDL cholesterol decreased in maternal plasma between Days 35 and 95 (P<0.01). On the other hand, there were no significant differences in plasma concentrations of fructosamine or triglycerides.

**Table 1:** Effects of nutritional restriction on maternal weight, adiposity and metabolism. Changes over time of pregnancy in maternal mean values (± S.E.M.) for body-weight, back-fat depth, and parameters of glucose and lipid metabolism.

	DAY 35	DAY 95
**Body-weight (kg)**	163.3±2.6	165.4±3.8
**Back-fat depth (mm)**	42.8±2.3	39.0±0.3.6
**Glucose (mg/dl)**	81.5±3.6^a^	61.2±5.9^b^
**Fructosamine (mg/dl)**	215.2±4.1	207.0±8.3
**Triglycerides (mg/dl)**	30.0±2.9	34.0±2.8
**Total cholesterol (mg/dl)**	94.0±4.9^a^	54.1±3.9^b^
**HDL-cholesterol (mg/dl)**	29.4±0.8^a^	10.4±2.1^b^
**LDL-cholesterol (mg/dl)**	58.6±4.6^a^	36.9±3.5^b^

Different superscript letters indicate significant differences among groups (P<0.01).

### Sex-related differences in fetal development

The total number of fetuses was fifty-one; a mean number of 8.7±0.6 fetuses/sow. Thirty-one fetuses were females, a higher percentage than males (60.8 vs 39.2%). Eight of the fifty-one fetuses (15.7%) were considered to have a severely compromised growth on the basis of one SD under the mean body-weight of the sampled fetuses; the percentage was lower in female than in male fetuses (12.9 vs 20.0%). However, the markers of fetal stress (cortisol and hydrogen peroxide) were not affected by sex or by severity of the growth restriction.

The comparison of absolute weights of male and female fetuses (Table 2) showed that male fetuses had higher total body-weight and carcass-weight than females (P<0.05 for both). On the other hand, females showed a higher relative weight of the brain when compared to the weight of the entire body and specifically of the head (P<0.05 for both). Similarly, the relative weights of total viscerae, liver, intestine, kidneys and spleen compared to both total body- and carcass-weights were also higher in females than in males (P<0.05 for all).

**Table 2:** Effects of sex on fetal morphometric parameters. Mean values (± S.E.M.) for body-size and weight of different structures and organs.

	FEMALE	MALE
**Body length (cm)**	33.8±0.3	34.2±0.4
**Occipito-nasal length (cm)**	8.8±0.1	8.7±0.2
**Biparietal diameter (cm)**	4.2±0.0	4.2±0.1
**Thoracic circumference (cm)**	18.8±0.2	19.5±0.3
**Abdominal circumference (cm)**	16.2±0.4	16.5±0.5
**Body weight (g)**	776.7±15.3^a^	828.8±18.7^b^
**Head weight (g)**	174.0±2.9	184.1±4.4
**Trunk weight (g)**	453.4±10.6^a^	488.1±11.8^b^
**Brain weight (g)**	24.8±0.4	24.3±0.5
**Heart weight (g)**	7.1±0.2	7.2±0.3
**Liver weight (g)**	23.0±0.6	23.0±0.8
**Intestine weight (g)**	39.4±1.1	38.6±1.4
**Kidneys weight (g)**	7.9±0.2	7.6±0.4
**Spleen weight (g)**	1.4±0.0	1.4±0.1
**Adrenal glands weight (g)**	1.3±0.9	1.4±0.1

Different superscript letters indicate significant differences among groups (a≠b, P<0.05).

Fetuses with severe growth retardation, independently of sex, were smaller in size and had lower absolute weights of head, carcass and total viscerae. The assessment of the ratio between head- and total body-weights showed no sex-related effects in these fetuses, with higher values in both male and female than in their littermates (P<0.0001). Similar results were found when comparing the brain-weight with both the head-weight and the total body-weight (P<0.0001 for both). Conversely, the relative weights of both carcass and total-viscerae related to total body-weight were affected by interactions between fetal development and sex (P<0.05 and P<0.01, respectively), being both values lower in fetuses with severe retardation, and lower in female fetuses.

The absolute weight of all the organs was lower in fetuses with severe retardation than in their littermates, excepting the spleen, which was around 30% heavier (P<0.05), independently of fetal sex. In consequence, all the fetuses with severe retardation, males and females, had higher relative weight of spleen to entire-body and total-viscera (P<0.01 for both). Conversely, the relative weight of liver and intestine was also affected by interactions between fetal development and sex (P<0.05 for both organs) with the lowest values being found in males with more severe retardation.

### Sex-related differences in fetal metabolism

Parameters related to glucose and lipid metabolism in fetal plasma and allantoic and amniotic fluids were similar between male and female conceptuses (Table 3). In case of growth retardation, both male and female fetuses showed an increase in glucose concentration at the amniotic compartment (P<0.005 and P<0.0005, respectively) but females also showed increased concentrations of cholesterol when compared to littermates with less severe restriction (P<0.05 for both parameters).

**Table 3:** Effects of sex on fetal metabolic parameters. Mean values (± S.E.M.) for parameters of glucose and lipid fetal metabolism.

		FEMALE	MALE
**Glucose (mg/dl)**	**Fetal blood**	83.0±14.5	78.4±26.1
	**Allantoic fluid**	11.3±1.2	9.5±1.0
	**Amniotic fluid**	9.0±0.9	9.0±0.9
**Fructosamine (mg/dl)**	**Fetal blood**	81.5±5.4	90.5±6.0
	**Allantoic fluid**	86.4±18.3	81.5±49.0
	**Amniotic fluid**	56.0±4.3	51.2±6.8
**Triglycerides (mg/dl)**	**Fetal blood**	22.3±2.2	23.0±3.1
	**Allantoic fluid**	7.9±1.8	4.5±0.8
	**Amniotic fluid**	4.0±0.6	5.0±1.0
**Cholesterol (mg/dl)**	**Fetal blood**	27.9±2.2	24.6±2.6
	**Allantoic fluid**	5.4±1.4	3.0±0.6
	**Amniotic fluid**	2.0±0.3	2.3±0.5

## Discussion

The nutritional level offered in the present study affected the normal pregnancy-related increases in maternal body-weight, similarly to previous trials [22], and decreased the maternal availability of glucose and lipids, which are especially critical for maintenance of an adequate intrauterine environment and, consequently, for viability and adequate growth of the conceptuses in swine genetically predisposed to obesity [36].

The response of the offspring to such negative environmental conditions was unequivocally driven by its sex, in spite of similar markers of metabolic and oxidative stress (glucocorticoids and hydrogen peroxide) in both sexes. There is a scarcity of studies on the effects of malnutrition on fetal oxidative status [37], but we can underline that the levels of circulating glucocorticoids have been found to be increased in rodent and primate offspring exposed to maternal undernutrition [38, 39]. Moreover, a previous study of our group addressed, coincidentally with other species, higher cortisol concentrations in purebred Iberian newborn females exposed to similar maternal undernutrition to the current trial [23]. The lack of differences in the fetuses of the current study, although we cannot leave aside genotype-related effects, may be related with gestational age; the final maturation of the hypothalamic—pituitary—adrenal axis is reached later, such that cortisol concentrations in fetal blood could change significantly just one week before parturition [40].

In the present study, female conceptuses showed higher viability rates, as evidenced by a biased percentage of female fetuses, and lower incidence of severe growth retardation, although differences were not found statistically significant in agreement with previous studies in purebred Iberian newborns exposed to maternal undernutrition [22, 23]. On the other hand, differences in developmental patterns between male and female fetuses were evident and significant; indicating protective strategies for the adequate growth and postnatal survival of the female individuals, coincidentally with previous data on the same swine pure breed [24]. In brief, both male and female fetuses prioritized brain development, but female fetuses were still able to maintain the development of other viscerae than the brain (mainly liver, intestine and kidneys) at the expense of the development of their own carcass.

Prioritization of brain development is a consequence of the “brain-sparing effect” [41]. Brain-sparing is a well-known mechanism for assuring the supply of oxygen and nutrients to the brain and, hence, the adequate development of this organ in case of IUGR [42–44]. In agreement, in the current study, brain-sparing was even more evident in the offspring affected by severe growth retardation, independently of sex; both male and female IUGR fetuses still favored brain development even when they were unable to maintain the growth of other viscerae. An adequate brain development assures, in turns, critical functions of the neonate such as breathing, suckling and other so-called autonomic functions [45] and, hence, assures or at least enhances vitality and probability of survival of the neonate.

Maintenance of the development of the liver and the intestine is also of paramount importance for neonate survival and postnatal growth [46]. Specifically, a “liver-sparing effect” in addition to the brain-sparing effect has been described in fetuses undergoing adverse conditions [47]. The adequate development and functionality of the liver assures glycogen deposition and gluconeogenic ability; which are essential during the first stages of the postnatal life, during the nutritional transition from continuous maternal supply of nutrients via the placenta to the intermittent supply from the mother milk via the intestine [48–50]. Concurrently, the adequate development of the intestine at this stage improves neonatal absorption and utilization of nutrients and other substances like immunoglobulins [51].

The renal system is known to be deeply affected by maternal undernutrition and IUGR, with a reduction in the number and functionality of nephrons and a decrease in the surface area of the glomerular capillaries which are the early causes for postnatal renal and cardiovascular (hypertension) diseases [52, 53]. Early experimental studies in swine have shown that fetuses are able to develop a compensatory hypertrophy in response to maternal undernutrition for maintaining renal function after birth [54]; this hypertrophy was more evident in the female fetuses of the current study.

On the other hand, the lack of effects of maternal undernutrition on spleen growth in both male and female offspring with severe growth retardation in the current study reinforces previous hypothesis about a more intense and earlier effect of IUGR on liver and kidneys than on spleen, with studies in humans evidencing a graded age-related effect on spleen development in which its growth is only affected at late pregnancy [55].

In the present study, the morphometric evidences supporting a better adaptation of female fetuses to maternal undernutrition are reinforced by differences in nutrient availability. There were no significant differences in glucose and lipid metabolisms between male and females either in fetal flood or allantoic and amniotic fluids. In case of severe growth retardation, both male and female fetuses showed an increase in glucose concentration at the amniotic compartment but females also showed increased cholesterol. Glucose is the main energy source for developing fetuses [56] while cholesterol is the key constituent of cell membranes and the precursor of hormones and metabolic regulators [57, 58]; hence, a high availability of cholesterol is essential for the viability and development of the fetus [59, 60]. The amniotic fluid components reflect dynamic exchanges between the fetus and the mother because it is derived from both the fetus and the mother [61] and we have previously found that fetuses from purebred Iberian dams with high viability rates and high placental efficiency have a high availability of glucose and cholesterol in the amniotic sac [36].

These results, although more specific studies are needed, are aligned to the current debate about whether the better survival and developmental traits of female offspring are due to themselves because of a better adaptive response of female conceptuses or due to a different maternal investment depending on the sex of the fetus. The Trivers—Willard hypothesis postulates that the external environment is signaled to the conceptus by the mother via the uterine environment and such signaling influences the phenotype of offspring produced to maximize chances of species survival [8]. Molecular signaling would be related to differences between sexes in the response to maternal corticosteroids and sex steroids and in the proper secretion of steroids by the offspring [25, 62].

In conclusion, the current study supports the existence of sex-related differences in morphometric patterns and metabolic pathways in case of mixed-race and nutritional challenge in swine, with better traits in female offspring. The evidences found about possible differences in maternal transfer and fetal availability of nutrients between sexes set the basis for further studies aiming to increase the knowledge on the interaction between genetic and environmental factors in the determination of adult phenotype in this swine model, which may be of translational value for subsequent studies in humans.
